# Rethinking referral pathways: qualitative evaluation of general practice networks to increase access to intrauterine contraception

**DOI:** 10.1093/fampra/cmac040

**Published:** 2022-04-29

**Authors:** Sara F E Bell, Caroline Harvey, Fiona Mack, Stephen Lambert, Mattea Lazarou, Kay Strom, Judith A Dean

**Affiliations:** Faculty of Medicine, School of Public Health, The University of Queensland, Herston, Queensland, Australia; Iris Education, Eldridge Street, Toowong, Queensland, Australia; Iris Education, Eldridge Street, Toowong, Queensland, Australia; Iris Education, Eldridge Street, Toowong, Queensland, Australia; Faculty of Medicine, School of Public Health, The University of Queensland, Herston, Queensland, Australia; Iris Education, Eldridge Street, Toowong, Queensland, Australia; Faculty of Medicine, School of Public Health, The University of Queensland, Herston, Queensland, Australia

**Keywords:** family planning, general practice, long-acting reversible contraception, pregnancy, Primary Health Care, reproductive health

## Abstract

**Background:**

Long-acting reversible contraceptives are recommended first-line contraception; however, intrauterine device (IUD) uptake remains low in Australia.

**Objectives:**

To describe the outcomes of an independent evaluation of the General Practitioner IUD Insertion Network (GPIIN), a project designed to address access barriers through formalized referral pathways between general practitioners (GPs) inserting IUDs and noninserters.

**Methods:**

An independent qualitative pragmatic inductive evaluation, involving 14 in-depth interviews with GPIIN members, was conducted 18 months post-GPIIN implementation in 2 Australian jurisdictions to identify and explore critical success factors and limitations of the model.

**Results:**

Local GP-to-GP IUD referral networks were considered a useful model to assist affordable and timely IUD access, improve noninserters’ IUD knowledge and inserters’ reflection on best practice. However, pathway simplification is needed to determine optimal integration of the concept into pragmatic GP-to-GP referral arrangements.

**Conclusions:**

GPIIN provides an opportunity to improve IUD access in Primary Health Care. Further consideration of organizations best positioned and resourced to facilitate sustainable delivery and coordination is necessary.

**Key messages**

- Formalized GP-to-GP referral networks have the potential to address known barriers to IUD insertion.
- Networks like GPIIN can improve access to affordable and timely IUD insertion.
- GP referral networks increase awareness of IUD suitability among noninserters.
- Networks like GPIIN can foster sharing of IUD knowledge and experience.
- Linking GP IUD inserters with other GPs can promote broader LARC knowledge.
- Research exploring effective network facilitation and support models is needed.

## Introduction

Long-acting reversible contraceptives (LARCs) are widely recommended as first-line contraceptive methods in Australia.^1^ However, despite known efficacy in reducing rates of unintended pregnancy,^2,3^ and increased prescription of levonorgestrel intrauterine devices (IUDs),^4^ uptake of LARCs, including insertion of IUDs, remains low.^5^ Contraceptive management is a core component of Primary Health Care (PHC) in Australia.^6^ However, general practitioners (GPs) are less likely to recommend IUD insertion compared with other contraceptives.^6^ Commonly cited barriers that limit GPs recommending and prescribing IUDs include misconceptions about nulliparous women’s eligibility,^7,8^ a belief that IUD insertion is outside GPs’ scope of practice, and expensive and time-consuming clinical training requirements to become an inserter.^8^ Recent studies suggest increasing numbers of GPs are becoming aware of the benefits and safety of IUDs.^5,9^ Nonetheless, access to IUD training programmes and suitably skilled GP inserters remains a major barrier to wider IUD uptake. Innovative models are needed to facilitate provision of IUD insertion as part of comprehensive contraceptive care within PHC settings. One such innovation is the development of formalized local GP-to-GP referral pathways.^10^

The General Practice Intrauterine Device Inserter Network (GPIIN) project developed by Iris Education, a Royal Australian College of General Practitioners (RACGP), and Australian College of Rural and Remote Medicine (ACCRM) accredited education provider in Queensland, Australia specializing in sexual and reproductive health (SRH) training for health practitioners,^11^ was a pilot project designed to address barriers to accessing IUD insertions. Through the establishment of formalized referral pathways between GPs inserting IUDs and noninserters, the model was designed to be suitable for upscaling to other geographic locations across Australia. The GPIIN project was supported by an Unrestricted Education Grant from Bayer Australia Ltd but the concept and all supporting resources were developed by Iris Education independent of any input from Bayer Australia Ltd.

**Fig. 1.**
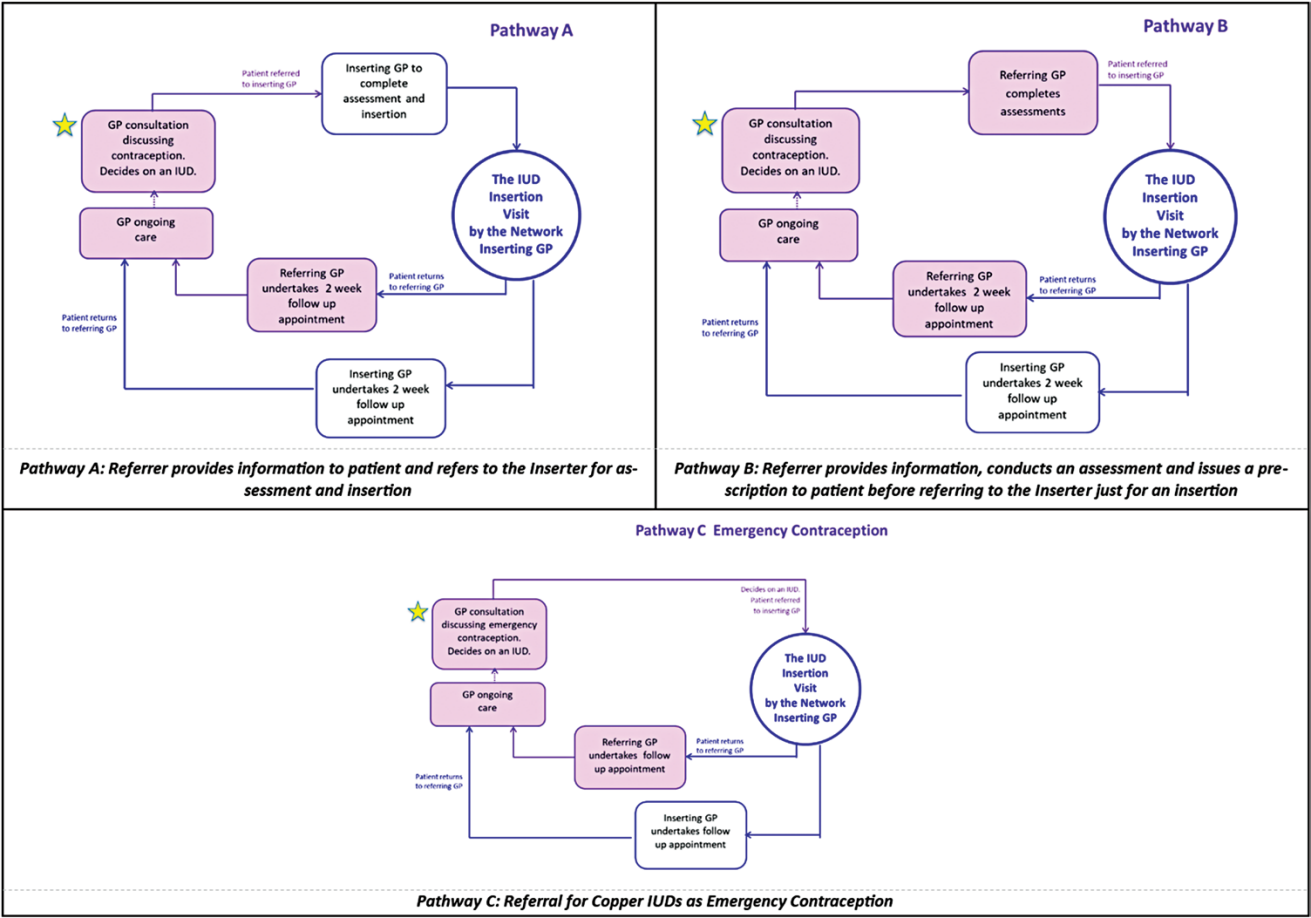
GPIIN referral pathways.

The GPIIN project proposed 3 referral pathways (Fig. 1). Pathways A and B recognize the inserting GPs’ responsibility for informed consent and patient care while allowing flexibility for the inserter to determine their preferred pathway based on the referring GPs’ skill in providing preinsertion assessment and information (Pathway A, skilled referring GP; Pathway B, less skilled referring GP). Pathway C was specific to Emergency Contraception (EC) copper IUDs and was developed in recognition of the limited use, despite its efficacy.^12,13^ All pathways guided patients back to the referring GP. The formalized referral pathways aimed to (i) create effective IUD access pathways; (ii) facilitate communication and trust between inserters and the referring GPs; (iii) minimize documentation time by providing referral letter templates and information leaflets for modification as required to be incorporated into GP practice software; and (iv) develop a community of practice to support peer education.^14,15^ A “Resource Kit,” including GPIIN tools and templates (developed through the Iris educators’ collective knowledge of best practice and clinical experience in specialized SRH and GP settings) plus a range of publicly available national and international evidence-based guidelines (Table 1) was provided in hard copy and electronically on a preloaded USB drive to GPs during network orientation to support implementation of the referral pathways. The “*Information and Counselling Guide*” and “*Preparing for your IUD*” tools provided referring GPs with structured support to ensure appropriate assessment and preparatory information prior to referral.

**Table 1.** Resources and tools provided to the GPIIN members during network orientation.

Resources provided to GPs during network orientation
1. Family Planning NSW Fact Sheets for Copper Mirena, 2013.
2. Family Planning NSW Fact Sheets for Copper IUD, 2013.
3. Family Planning Alliance Australia LARC Position Statement, 2014.
4. Family Planning Alliance Australia: LARC Efficacy Statement, 2014.
5. Family Planning Alliance Australia: Emergency Contraception Family Planning New South Wales, Family Planning Victoria and True Relationships and Reproductive Health. Contraception: An Australian Clinical Practice Handbook, 4th edition. Family Planning New South Wales, Family Planning Victoria and True Relationships and Reproductive Health 2016. Chapter 2: Intrauterine Devices.
6. Faculty of Sexual and Reproductive Healthcare Clinical Effectiveness Unit. FSRH: Clinical Guidance: Intrauterine Contraception, 2015.
7. The Faculty of Sexual and Reproductive Healthcare Clinical Effectiveness Unit. FSRH: UK medical eligibility criteria—Summary Sheet, 2016.

Tools and templates provided to GPs during network orientation
1. GPIIN: IUD information and counselling guide (Pathways A and B)
2. GPIIN: Referral letter to inserter (Pathways A and B)
3. GPIIN: Preparing for your insertion (Pathway B)
4. GPIIN: Return letter from inserter to referrer (Pathway A and B)
5. GPIIN: Post-IUD insertion patient letter

The GPIIN project commenced in April 2017 with circulation of project information to GPs by pharmaceutical representatives from Bayer Australia Ltd. Fifty-seven GPs (22 inserters, 35 referrers) expressed interested and were invited to attend face-to-face project orientation meetings independently facilitated by the Iris Education team. The first meeting, for experienced inserting GPs, introduced the GPIIN concept. The referral pathways (Fig. 1) and “Resource Kit” (Table 1) were reviewed by the GPs attending this meeting and refined prior to implementation by the Iris Education team. Twenty (11 inserters, 9 referrers) of the 57 (35%) interested GPs went on to be actively involved with one of the 4 networks established, 3 in South-East Queensland (SEQ) and 1 in northern New South Wales (NSW). A second project orientation meeting attended by both inserting and referring GPs was held in each network. This subsequent meeting had 3 aims: (i) introduce referring GPs to the GPIIN concept, (ii) increase knowledge and dispel misconceptions about IUDs, and (iii) establish collegiate relationships between network members to build confidence in the localized GP-to-GP referral process. The orientation meetings provided opportunity for discussion of specific preferences for referral procedures between attending referrers and inserters (i.e. using established communication processes, reviewing use of the provided referral letter template, patient-initiated appointment). However, the intent of the network was to facilitate relationships that enabled GPs to have control of a pragmatic referral process to fit with their own practice, thereby leaving the ongoing use of tools and chosen pathways up to the network members to decide and communicate between themselves.

This paper reports the findings from an independent evaluation of the GPIIN project conducted approximately 18 months after implementation. The objective of the GPIIN evaluation project was to explore the “real world” experiences and perceptions of the network members and identify the enablers, barriers, and critical success factors in the implementation and ongoing management of GPIINs. Outcomes of this evaluation provide recommendations for planning and supporting future GPIINs.

## Methods

### Study design

A qualitative pragmatic inductive evaluation approach involving semistructured interviews was applied to allow exploration of the nuance experiences of the GPIIN members.^16^ The GPIIN evaluation project was conducted over 2 months from November 2018 by 2 experienced SRH researchers (SB and JD). The GPIIN evaluation was independently funded by Iris Education, Bayer Australia Ltd was not involved in the evaluation and had no influence on the outcomes. The researchers both had clinical experience in providing SRH care including provision of contraception and were known to Iris Education but did not know any GPIIN members interviewed and had no relationships/activities/interests with Bayer Australia Ltd or other pharmaceutical company involved with the manufacture of IUD or other contraceptive methods. The research evaluation team was responsible for all data collection, management, and analysis.

The independent evaluation was conducted under The University of Queensland Human Research Ethics Committee ethical approval (Approval Number #2018001958). The GPIIN evaluation research was undertaken with appropriate informed consent of participants.

### Recruitment

Information about the opportunity to be interviewed as part of the evaluation was circulated to all network members by the GPIIN developers on joining the GPIIN project. Convenience sampling^17^ was applied to recruit participants from the 20 GP network members who provided written consent on joining the GPIIN project to be contacted by the UQ evaluation team. All network members who agreed to be interviewed were interviewed. Pharmaceutical representatives involved with circulating GPIIN study information and key stakeholders involved with setting up interstate networks based on the GPIIN model were also offered opportunity to join the evaluation to allow for broader exploration of the network concept and processes.

### Data collection

Approximately 20–30 min in length, the digitally recorded interviews were conducted face-to-face in a private consultation room at the interviewees place of work or via teleconference. The interview guide was developed by the researchers (JD and SB) and collaboratively refined with Iris Education team (CH, FM, SL, and KS) to ensure clarity of questions relating to specific meeting elements e.g. resource pack inclusions and meeting types. Sample size was limited by time and funds for renumeration. All participants were offered a $150 gift card as compensation for their time on interview conclusion. The researchers debriefed after each interview to reflect on data collection, discuss and share ideas about evolving themes in preparation for subsequent interviews.

### Data analysis

NVivo 12 (QSR International Pty Ltd, 2018) was used to store and manage data during the thematic analysis of deidentified verbatim transcribed interviews data conducted by 2 researchers (JD and SB) using the following steps: proof reading for transcription accuracy, reading and familiarization, coding, searching for themes, reviewing themes, defining and naming themes, and finalizing the analysis.^18^ The researchers (JD and SB) independently coded all interviews. The codes were articulated into themes by JD in collaboration with SB, any inconsistencies or discrepancies were discussed and resolved by JD, SB, and ML. Content analysis of the deidentified field notes collected by the Iris Education team during GPIIN implementation and follow-up telephone calls with GPIIN network members approximately 3 months after each GPIIN was established were also conducted by the 2 researchers (JD and SB). Findings of the content analysis were used to provide background for the researchers and understanding to inform recommendations. On conclusion of the independent evaluation data analysis, the final codebook, with deidentified excerpts from the transcripts supporting key themes, was presented to the Iris Education team (CH, FM, SL, and KS), to guide development of recommendations in collaboration with the researchers (JD, SB, and ML).

## Results

### Participant characteristics

Five GP inserters, 2 referrers, 1 pharmaceutical representative, 3 Iris Education team members, and 3 stakeholders involved with establishing and facilitating interstate networks (*N* = 14) were interviewed as part of the independent evaluation. Four of the 5 GP inserters had been inserting IUDs for between 6 and 12 years, 1 had commenced inserting in the previous 12 months. The 3 stakeholders were all GPs inserters with expertise in providing SRH clinical and IUD insertion education, and, while they were not involved with the development or implementation of GPIIN in Queensland, they were involved with GPIIN interstate roll-out and provided additional insight that could help inform future expansion into other locations. Table 2 outlines IUD access barriers identified by the interviewees.

**Table 2.** Barriers to accessing IUDs identified by GPIIN General Practitioners.

1. Cost to GPs to provide IUD insertion services including IUD insertion training, loss of income due to training requirements, poor Medicare remuneration comparative to required investment into specialized skills and equipment.
2. Cost to the patient associated with referral to specialist services.
3. Limited knowledge of IUDs among women, GPs, and other health care professionals.
4. Women’s personal and family/friends/acquaintance experiences of bleeding and weight gain associated with IUD and other hormonal contraception, particularly hormonal implants.
5. Myths and fears generated by the media, bloggers, family, and friends and lack of knowledge. Examples given included: fear of the procedure, fear of something “foreign” inside, multigravida women thinking it will fall out, and a belief by women and GPs that nulliparous women are not able to use an IUD.
6. Limited access to GP inserters.
7. GPs not recommending IUD as a contraception choice.
8. Reluctance of both women and GPs to refer to specialists due to associated expense, delays, and time.
9. GPs unaware of what services other GPs in their area are offering and to whom to refer women for IUD insertion.
10. Access to suitable infrastructure and staffing within the practice setting to support IUD insertion, such as single GP practices with no practice nurse or suitable treatment room/area to safely perform procedures.

### Rationale for GPIIN involvement

Participation in the GPIIN project was driven by increasing demand for IUDs within their practices, a need to address access barriers, streamline IUD service provision, increase IUD knowledge, and broaden the scope of GP’s practice. IUD insertion was considered a key responsibility and skill required by GPs.

Insertion of IUDs, I don’t believe, sits in a specialist role. It’s a skill that a GP should be able to do. (Inserter)

The opportunity to meet and share experiences with like-minded GPs interested in SRH, was also considered a valuable outcome, as busy schedules often prevented networking.

I think the biggest thing is that it’s helped networking, helped GPs to sort of come out from under their hidey holes, to share their knowledge and experience with others. (Network Facilitator)

### Perceptions of the GPIIN orientation meetings

The network orientation meetings with embedded educational component were considered essential but it was important that this be tailored to differing IUD knowledge and experience levels among inserters and referrers and incorporate information on comprehensive contraception options.

I thought I would have seen more about the actual process of insertion… a little more about technique. (Referrer)

### Perceptions and experiences of the GPIIN model

The evaluation results identified 5 key themes in regards to the enablers, barriers, and critical success factors of the GPIIN concept: (i) Networks have the potential to increase women’s IUD access; (ii) Formalized GP-to-GP referral process and foster collegial trust and limits potential for loss of patients; (iii) GPIIN concept and referral pathways were useful but too complex; (iv) Pathway preferences and usage depended on GP preference and experience; and (v) GPIIN Facilitators are essential for sustainability and growth.

#### Networks have the potential to increase women’s IUD access

Interviewees noted that the GPIIN project addressed barriers to IUD access in the PHC setting by increasing GP knowledge and confidence providing IUD information and referral, particularly for young, nulliparous, and perimenopausal women. Waiting times were also reduced through negating the need for specialist referral. However, there were disparate thoughts on whether the model had increased access, with most inserters unable to recall receiving any referrals via GPIIN pathways.

In an ideal world, there would be no routine insertions happening in hospital outpatients, it could be kept free for complex [cases], and others would be done in a GP-based setting (Network Facilitator)

#### Formalized GP-to-GP referral process fosters collegial trust and limits potential for loss of patients

The potential loss of patients to other GPs raised some concerns. However, many participants considered formalizing the referral networks fostered trust, confidence, and collegiality between GPs and dispelled “*patient poaching*” concerns. Both inserters and referrers commented that they were “*so busy now*” that they did not need to “*poach*” patients.

I’m busy, I’m chock a block with appointments, I don’t want to keep patients for anything extra. I do want to discharge them back to their normal GP. (Inserter)

I’ve no concerns about outsourcing to another GP with expertise…I’m so busy…. not hungry to keep clients (Referrer)

#### GPIIN concept and referral pathways were useful but too complex

Overall, the GPIIN model was considered flexible enough to adapt to differing clinics’ practices and the proposed referral pathways were useful; however, it was all unnecessarily complex. Several participants suggested that a single pathway would be more streamlined.

the network is a definite necessity, but for easy flow and access, it’s about making the network as simple as possible….. it was you can do it this way, or you can do it this way. I suppose some GPs might prefer one to another, but that was a wee bit confusing, having option A, option B, etcetera. (Inserter)

found it a bit clunky…. filling out a form became more work, whereas found it easier to say to the patient, this is the doctor I’m going to refer you to, give them a call or ask the receptionist to give them a call. (Referrer)

#### Pathway preferences and usage depended on GP preference and experience

Referral pathway selection was influenced by the experience, time constraints, and preference of the GPs, particularly the inserter. Most considered Pathway A preferable wherein the inserter completed the clinical assessment.

I think the GP inserters overwhelmingly want to do the pre-insertion [assessment] themselves [Pathway A], because the buck stops with them…for example, had I perforated the uterus….. [and] hadn’t explained that risk to the patient, I would feel that I was negligent. (Network Facilitator and Inserter)

Adopting Pathway B, wherein the referrer provides information, conducts an assessment, and issues a prescription to the patient before referring for insertion, was conditional upon whether the inserter knew and trusted the referring GP’s knowledge and skills.

Some of my colleagues, who are very good at [IUD assessment] …I do accept [Pathway B]. They [referrer] usually call me and say I’ve done this, this, this, are you happy to see them? … Some patients, I would rather do a full assessment first [Pathway A], because as an inserter, I hold my responsibility to take a patient. (Inserter)

No interviewees could recall using Pathway C, despite their increased “*confidence in the copper IUD*” and being provided one at the project orientation workshop.

#### Resources were good but not used

Several interviewees could locate their resource kit, however, most reported not using the kit and no one reported integrating the GPIIN template tools into practice software. Many believed that the paperwork was:

too complicated and unnecessary especially under time pressure and always someone waiting, [I am] too old to learn new practices when [a] simple referral letter system is working. (Referrer)

#### GPIIN Facilitators are essential for sustainability and growth

Having someone responsible for facilitating the network was considered essential for network sustainability and potential expansion of the concept. Some inserters described regular contact with their local network facilitators, and one referrer described “*above and beyond*” ongoing support. However, others described having minimal contact with network facilitators following the initial face-to-face network orientation meeting. Pharmaceutical representatives acted as network facilitators, as frequent contact with GPs is a core part of their professional role, however, most of the GPs considered pharmaceutical representatives as network facilitators a potential conflict of interest. Primary Health Networks (PHNs), independent organizations responsible for encouraging GP collaboration and service delivery planning in allocated regions,^19^ were identified as potential independent facilitators. However, some participants perceived that SRH was considered a low priority by their local PHN and that this would impact on their capacity to allocate support for GPIINs. GP inserters with SRH specialist training were suggested as ideal network facilitator candidates. However, some interviewees felt that GPs promoting themselves within the network and subsequent competition for patients might also create potential conflict of interest.

People see them [Pharmaceutical representatives] as being biased… you know they’re there just to sell a product…an ideal rep would be getting either an inserter, or someone [GP] from Family Planning, to go around to practices, but that’s all about time and cost. (Inserter)

The lack of Practice Manager involvement in the GPIIN project was identified as a key barrier to the expansion and success of networks. Field notes suggest Practice Managers were generally unaware GPs within their practice were involved in the project. This lack of awareness resulted in them acting as “*gate keepers*” preventing follow-up support communication by the Iris Education team. Subsequently, practice administration staff were not informed about the project and where thus unprepared to provide information to patients enquiring about IUD insertion nor advised to incorporate GPIIN resources into practice software.

## Conclusions

The GPIIN project is one of the first to develop, document, and evaluate a model for implementing local GP-to-GP referral pathways designed to increase access to IUD insertion in PHC settings. Data collected during the project implementation does not provide sufficient evidence to determine if IUD insertion increased. Nevertheless, the independent evaluation suggests that formalized referral networks, linking IUD inserters and noninserters within local areas, has potential and in-principle support from key stakeholders. However, further research is warranted to fully understand how they can be used to their full potential to address the service and patient barriers to IUD access identified in this study and previous literature.^20–23^

The GPIIN model, combining education and upskilling, has the potential to provide inserters the opportunity to maintain clinical currency and competency, while creating a community of noninserting GPs with the knowledge, skill, and mechanisms to provide their patients with access to IUDs. Such networks reduce the need for referral to specialists or public hospitals’ waiting lists, thereby reducing delay to insertion, minimizing the inherent risk of unintended pregnancy while using less effective contraception, and decreasing the economic burden created by the need to use private health services or other forms of more expensive contraception.^24^ There was consensus among those interviewed that IUD insertions can successfully occur in PHC settings and, consistent with previous research,^5,8^ this study reinforces that IUD insertion should be considered a core component of contraceptive management for GPs, particularly as patient driven demand for IUD insertion increases.

Consistent with previous studies,^8^ formalized referral pathways were considered a “*necessity*,” but require ready mechanisms to sustain communication between the inserter and referrer in order to build trust and ongoing circular sharing of knowledge.^14^ The GPIIN resource kit was comprehensive, however, infrequently used, with referrers citing preference for their own familiar resources. This calls for scoping of existing practice processes when setting up local networks to identify existing systems that can be modified. The evaluation also highlighted that the process of engaging directly with the GP to establish the network limited involvement of other practice workforce that may have assisted in sustaining communication and increasing referrals. For example, including Practice Managers from initiation may have offered a catalyst for raising awareness of GPIIN among their practice staff and patients and other GPIIN practices.

Network facilitation also requires further consideration, specifically selection of the most appropriate institutions or individuals for this key role. The successes observed within functioning GPIINs largely relied on the enthusiasm, and dedication of individual GPs with an interest in contraception access. The initial inserter only meeting could be better utilized as the catalyst to identify local network leaders to take a more active facilitation role. GPIIN networks are unlikely to be sustainable without dedicated logistical and financial support. There was broad agreement that PHNs had the established infrastructure to support the expansion of the GPIIN concept and coordination of broader accessible SRH care.^25^ GP inserters may be well placed to be the network facilitators if the GPIIN concept is funded within the local established and trusted PHN Health Pathways platforms, as sustainable locally relevant referral pathways for GPs to implement.^26,27^ Additional work is required to determine the optimal network size (including referrer to inserter ratios) to maintain network momentum.

Insufficient data collection during implementation combined with logistical challenges with GP recruitment to GPIIN and the subsequent evaluation interviews limited project evaluation through under-representation of referrers despite considerable recruitment attempts, potentially resulting in unmet saturation of themes and recruitment bias. Nonetheless, findings from the independent qualitative enquiry of GPIIN provides valuable insights to inform future development of networks that are flexible and responsive to the increasing demand for IUDs.^4^ Future research should consider effective methods for GPs recruitment. The authors acknowledge the limitations of representativeness and bias resulting from convenience sampling, however at the pilot stage of enquiry economical methods were necessary and findings were not anticipated to be generalizable.^17^ For example, the level of engagement of different stakeholders within each network is likely to vary and further exploration of the suitability of pharmaceutical representatives acting as Network Facilitators is recommended. There were also no data collected within the GPIIN project that enabled the evaluation team to determine if the network had resulted in referrals, supporting the need for user friendly systems to record and report referral/referee information to quantify outcomes.

Access to IUD training programmes is noted as a major barrier to wider IUD uptake.^5^ The authors fully support the need to increase access to training that is affordable and designed to meet the GPs time constraints and needs,^8^ however, the scope of this project was not to directly increase the number of GP inserters. Rather based on recommendations from previous research,^5^ the project sought to increase referrals to existing underutilized skilled and willing local GP IUD inserters. By doing this the project also provided opportunity for existing GP inserters to maintain skills,^5^ increased access to IUD insertion, and support IUD service provision cost effectiveness by reducing the need for tertiary referrals.^5^

GPIIN offers a model which addresses some of the known service-related barriers to IUD uptake. Sexual and reproductive health care, including comprehensive contraceptive counselling and IUD insertion, is well within the scope of Australian GPs. Localized GP-to-GP referral networks can increase access to and uptake of IUDs, but further investigation on how best to integrate the network concept into pragmatic local GP-to-GP arrangements and provide the ongoing dedicated support needed to establish and maintain these networks is required.

## Supplementary Material

cmac040_suppl_Supplementary_Checklist

## Data Availability

Data available on request.
